# An online experiment to assess bias in professional medical coding

**DOI:** 10.1186/s12911-019-0832-x

**Published:** 2019-06-20

**Authors:** Jacqueline M. Torres, Danielle Hessler-Jones, Carol Yarbrough, Adam Tapley, Raemarie Jimenez, Laura M. Gottlieb

**Affiliations:** 1 Department of Epidemiology & Biostatistics, UC San Francisco, 550 16th Street, San Francisco, CA 94143 USA; 2 Department of Family & Community Medicine, 500 Parnassus Avenue, San Francisco, CA 94117 USA; 3 Telehealth Resource Center, UCSF Health, 350 Parnassus, 609A, San Francisco, CA 94117 USA; 4 UC San Francisco, 505 Parnassus Ave, San Francisco, CA 94143 USA; 5 American Association of Professional Coders, 2233 S Presidents Dr, West Valley City, UT 84120 USA; 6 Department of Family & Community Medicine, 1001 Potrero Ave, San Francisco, CA 94110 USA

## Abstract

**Background:**

Multiple studies have documented bias in medical decision making, but no studies have examined whether this bias extends to medical coding practices. Medical coding is foundational to the US health care enterprise. We evaluate whether bias based on patient characteristics influences specific coding practices of professional medical coders.

**Methods:**

This is an online experimental study of members of a national professional medical coding organization. Participants were randomly assigned a set of six clinical scenarios reflecting common medical conditions and asked to report encounter level of service codes for these clinical scenarios. Clinical scenarios differed by patient demographics (race, age, gender, ability) or social context (food insecurity, housing security) but were otherwise identical. We estimated Ordinary Least Squares regression models to evaluate differences in outcome average visit level of service by patient demographic characteristics described in the clinical scenarios; we adjusted for coders’ age, gender, race, and years of coding experience.

**Results:**

The final analytic sample included 586 respondents who coded at least one clinical scenario. Higher mean level of service was assigned to clinical scenarios describing seniors compared to middle-aged patients in two otherwise identical scenarios, one a patient with type II diabetes mellitus (Coef: 0.28, SE: 0.15) and the other with rheumatoid arthritis (Coef: 0.30, SE: 0.13). Charts describing women were assigned lower level of service than men in patients with asthma exacerbation (Coef: -0.25, SE: 0.13) and rheumatoid arthritis (Coef: -0.20, SE: 0.12). There were no other significant differences in mean complexity score by patient demographics or social needs.

**Conclusion:**

We found limited evidence of bias in professional medical coding practice by patient age and gender, though findings were inconsistent across medical conditions. Low levels of observed bias may reflect medical coding workflow and training practices. Future research is needed to better understand bias in coding and to identify effective and generalizable bias prevention practices.

**Electronic supplementary material:**

The online version of this article (10.1186/s12911-019-0832-x) contains supplementary material, which is available to authorized users.

## Background

A substantial body of work has demonstrated that implicit and explicit bias based on patient race/ethnicity, gender, and other social factors influences medical decision-making [1–10]. Studies have found that implicit bias against racial minorities is associated with inadequate pain treatment [11] and poorer quality care post myocardial infarction [12]. Other work has demonstrated similar disparities for female, elderly, and differently-abled patients, as well as patients experiencing homelessness [7–10]. Though the existing literature in this area has included physicians, residents, medical students, nurses, and a range of mental health professionals (psychologists, clinical social workers, counselors, graduate students), to our knowledge there has not been analogous research conducted on bias in medical coders. This gap in awareness around whether professional coder practices are subject to similar biases as those found in other medical disciplines is particularly relevant given the important role of coding in establishing the quality and cost of care.

Medical coding informs care delivery and referrals, population health initiatives, and payment [13], and remain fundamental even in the transition from fee for service to value-based models of care. While physicians are often tasked with assigning these codes, an increasing number of health systems use professional medical coders to review physician-assigned codes or to assign codes based on visit records [14]. Despite rapid expected growth in the professional medical coder workforce [15], there is little publicly available information about the share of coding done by professional coders, and no academic literature of which we are aware evaluating professional coding practices. This stands in contrast to other areas of medicine where standards for evidence-based practice are strongly encouraged.

The primary objective of this online experimental study with professional medical coders was to test for bias in the use of procedural codes based on patients’ demographic characteristics (e.g. race/ethnicity, age, gender, and ability) or documented social needs (e.g. homelessness, food insecurity). Given evidence suggesting that bias based on these factors affects other medical decision-making, we hypothesized that demographic and social information in these charts would influence professional coder decisions around interpreting and assigning level of service codes to sample clinical scenarios. Based on the existing literature, we expected that coders would assign different levels of service to clinical scenarios describing patients that were members of demographic minority groups or that had potentially stigmatizing social needs, as compared to otherwise identical charts that described patients from majority groups or those who had no documented social or economic disadvantages [1, 7].

## Methods

### Data

Data come from an online study of members of a national professional medical coding organization in the United States (US). In August 2017, a description of the study was included inside an emailed organization newsletter. Interested participants were sent a one-time invitation to click on an external link that led to the study website, where they could read further information regarding study objectives and inclusion criteria. Stated inclusion criteria included being of > 18 years of age, working at least 15 h per week as a certified medical coder in the US, and working with Evaluation & Management (E & M) Coding, following either 1995 [16] or 1997 [17] guidelines. We additionally asked that participants have experience coding within a general medical system setting (i.e. not only in a specialty or ED setting). Once on the website, coders consented to participate by clicking a link that brought them to an initial set of survey questions about their professional history and demographic characteristics. Screening and survey procedures were completed in the REDCap secure data environment. All study procedures were approved by the Institutional Review Board at the University of California, San Francisco.

### Experimental procedures

After completing initial survey items regarding work history and demographic background, respondents were subsequently randomized to one of six experimental arms, five of which were related to the present study about identifying bias around patient-level factors, including race, age, gender, ability, and social or economic need. Respondents randomized to a sixth arm were asked to complete a separate set of questions regarding the use of ICD-10 “social” Z codes. These questions solicited qualitative responses about using codes for patient scenarios and were not related to bias in medical coding; data on this sixth experimental arm is therefore outside the scope of the present study.

Each respondent was asked to read through six different medical visit scenarios reflecting patient visits for common medical conditions (newly diagnosed diabetes mellitus type 2, pharyngitis, rheumatoid arthritis, hypertension, asthma exacerbation, and chest pain) and to assign the most appropriate Current Procedural Terminology (CPT) code reflecting level of service for each scenario. These clinical scenarios were adapted from prior coder certification exam questions administered by the partnering professional membership organization; they were selected specifically to include some degree of uncertainty about the appropriate CPT code.

Each respondent was first randomized to one of six experimental arms aimed at assessing bias along a single dimension of patient demographics or characteristics (e.g. race, gender). Within each arm, respondents were further randomized to a sub-group in which they viewed an otherwise identical sequence of clinical scenarios that varied patients’ demographic or social and economic need characteristics. For example, a respondent might be randomized to the experimental arm evaluating age-related bias (the 2nd experimental arm) and then further randomized to subgroup A, in which the first three clinical scenarios described an older adult patient while the last three described a patient in young or mid-adulthood; OR to subgroup B, in which the last three clinical scenarios described an older adult patient, while the first three described a patient in young or mid-adulthood. (See Additional file 1: Table S2 for an overview of charts assigned within each experimental arm and sub-group).

### Measures

#### Work experience and demographics

Prior to randomization, respondents answered questions about years of experience and current hours per week working as a coder, age (in years), gender, and race/ethnicity. We collapsed the measure of race/ethnicity to a binary measure contrasting non-Latino white with racial/ethnic minority and/or mixed/biracial respondents, given the relatively small number of respondents identifying with the latter categories.

#### Level of service

For each medical chart, respondents were asked to select one CPT code from five possible standard level of service code choices [(e.g. 99,201 through 99,205 (new patients); or 99,211 through 99,215 (established patients)]. Level of service codes are intended to be calculated based on a presented history, physical examination performed, and complexity of medical decision-making related to the visit [18, 19]. Coder responses were recoded so that they ranged from one to five, with five generally reflecting the highest level of service.

### Statistical analyses

We first generated descriptive statistics for work and demographic characteristics across the overall sample and for each experimental arm, and tested for significant differences in these characteristics with t-tests and Chi-squared tests. Comparisons within each study arm were powered initially to moderate effect sizes for differences in level of service scores between sub-groups (standardized effects sizes between d = 0.58 to d = 0.54, translating to mean differences of 0.45 to 0.25 points on the 5-point level of service scale, with the range dependent on observed standard deviations for each experimental arm and clinical scenario). To increase statistical power, we repeated analyses after pooling respondents from across select study arms when their assigned charts presented identical patient demographic characteristics and health conditions. For example, for the first clinical scenario (asthma exacerbation), we pooled responses from all respondents assigned to view a chart that described an 83-year old female patient (see Additional file 1: Table S2). This pooling of respondents created larger comparison groups, which enabled us to achieve at least 80% power to detect standardized effect sizes ranging from d = 0.48 to d = 0.51 (translating to mean differences of 0.40 to 0.20 on the 5-point level of service scale).

We used Ordinary Least Squares regression to estimate adjusted differences in mean level of service assigned to charts within experimental arms, controlling for respondent age, gender, ethnicity, and years of coding experience. The results of these models were compared to results from models that re-weighted respondents with stabilized inverse probability weights (IPWs) that accounted for respondent attrition over the course of the survey (e.g. attrition from the demographic questionnaire to randomization; attrition after each subsequent clinical scenario) [20]. IPWs can help account for potential biases due to differential respondent attrition based on respondent demographic characteristics or other factors that might also influence assigned level of service. These IPWs including respondent demographic and work experience indicators were created with logistic regression models estimated on the 801 respondents who reported complete demographic and work experience information.

## Results

**Fig. 1:**
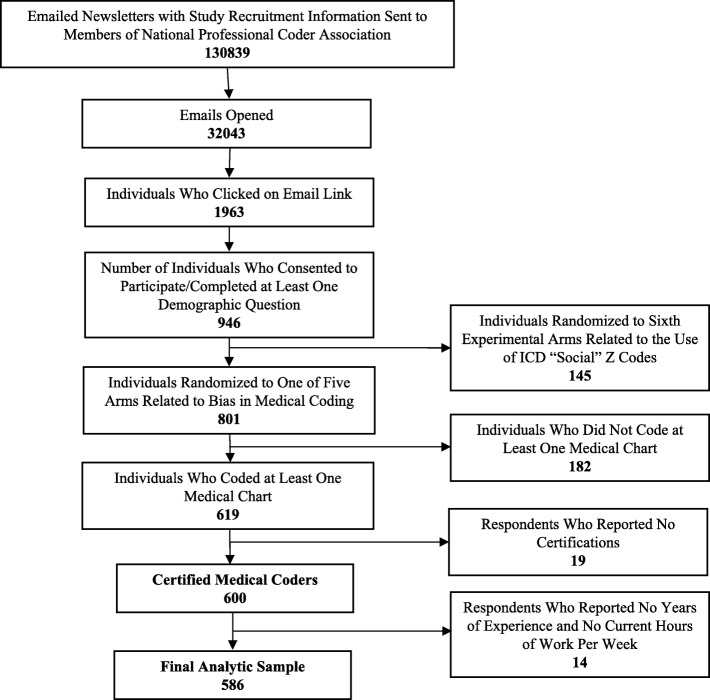
Flow Chart of Respondent Recruitment, Attrition, and Inclusion

The survey invitation was embedded in a newsletter sent to 130,839 listserv members; 32,043 opened the email and 1963 clicked on the link to participate in the survey (see Fig. 1). A total of 946 respondents answered at least one demographic survey question. For the present analyses, we excluded 145 respondents assigned to an experimental arm unrelated to the bias in medical coding study objective. We additionally excluded 182 respondents who did not complete the coding exercise for at least one medical chart, 14 respondents who reported both zero (0) years of work experience and zero (0) current hours/week as a professional medical coder, and 19 respondents who reported that they held no medical coding certifications. The final analytic sample includes 586 respondents with at least some experience as a professional medical coder who coded at least one medical chart.

Comparing the number of individuals who received the newsletter to those who completed at least one demographic survey item, the response rate for the study overall was 0.007%; the rate was 3.0% if we compare all study respondents who completed at least one survey item to those who opened the recruitment email. There was additionally respondent attrition from the demographic survey items to the experimental portion (22.7%). We attempted to address this attrition with inverse probability weighting. Respondents who coded at least one medical scenario reported more years of coding experience and more hours per week of coding work compared to respondents who did not code any medical scenarios.

The majority of respondents in the analytic sample identified as female (92.3%) and non-Latino White (81.5%); respondents were 45.6 years on average (SD + 11.2) (Table 1). Respondents worked as professional medical coders for an average of 11.4 years (SD + 8.9) and reported currently working for a mean of 37.7 h per week (SD + 11.4). There were no significant differences in respondent demographic and work characteristics across respondents in the experimental sub-groups and corresponding study arms considered in the present analyses (Additional file 1: Table S3).

**Table 1:** Overall Demographic and Work Characteristics for a Sample of Professional Medical Coders (*N* = 586)

Female, n(%)^a^	538 (92.3)
Non-Latino White, n (%)^b^	448 (81.5)
Age, mean (SD)^c^	45.6 (11.2)
Years worked, mean (SD)^d^	11.4 (8.9)
Hours worked per week, mean (SD)^e^	37.7 (11.4)

Source: Original data from an online experiment of professional medical coders in the US, August–September, 2017. ^a^ Out of *N* = 583 respondents with non-missing gender information. ^b^ Out of *N* = 550 respondents with non-missing race/ethnicity information. ^c^ Among *N* = 570 respondents with non-missing age information. ^d^ Out of *N* = 580 respondents with non-missing information on years worked. ^e^ Out of *N* = 577 respondents with non-missing information on hours worked per week

In models that contrasted outcomes among sub-groups within experimental arms (Table 2), higher mean level of service was assigned to seniors compared to middle-aged patients in two otherwise identical scenarios, one a patient with rheumatoid arthritis (Coef: 0.30, SE: 0.13, *p* < 0.05) and the other a patient with type II diabetes mellitus (Coef: 0.28, SE: 0.15, *p* < 0.10). Female patients were assigned lower level of service scores compared to male patients for medical scenarios describing asthma exacerbation (Coef: -0.25, SE: 0.13, *p* < 0.10) and newly diagnosed rheumatoid arthritis (Coef: -0.20, SE: 0.12, *p* < 0.10). There were otherwise no differences in average level of service for the remaining clinical scenarios, which included four additional scenarios related to age; four related to gender; six related to differences by patient race; six related to ability differences; and six reflecting differences in documented social need.

**Table 2:** Ordinary least squares regression models of level of service score (range: 1–5) assigned to six sample charts by professional medical coders, by patient demographic characteristics or social need^*^

	Chart 1		Chart 2		Chart 3		Chart 4		Chart 5		Chart 6	
	Coef	SE	Coef	SE	Coef	SE	Coef	SE	Coef	SE	Coef	SE
Experimental Arm 1 (Racial Bias)												
White patient (ref)												
African-American patient	−0.078	(0.13)	0.123	(0.15)	0.109	(0.14)	0.020	(0.13)	0.057	(0.15)	0.107	(0.15)
Experimental Arm 2 (Age Bias)												
Middle-aged patient (ref)												
Older adult patient	−0.006	(0.15)	0.284	(0.15)*	0.300	(0.13)**	−0.015	(0.12)	−0.140	(0.11)	−0.157	(0.23)
Experimental Arm 3 (Ability Bias)												
Patient without disabilities (ref)												
Patient with hearing/visual/physical disability	0.007	(0.13)	0.088	(0.13)	0.058	(0.12)	0.015	(0.12)	0.110	(0.12)	−0.020	(0.17)
Experimental Arm (Gender Bias)^†^												
Male patient (ref)												
Female patient	−0.250	(0.13)*	−0.073	(0.13)	−0.200	(0.12)*	0.143	(0.12)	−0.024	(0.13)	0.181	(0.17)
Experimental Arm 5 (Social Need)												
No social need (ref)												
Housing Insecurity	0.118	(0.13)	0.055	(0.13)	0.105	(0.09)	−0.107	(0.12)	−0.055	(0.11)	0.038	(0.17)
Food Insecurity	0.076	(0.13)	0.076	(0.12)	0.026	(0.09)	0.015	(0.11)	0.023	(0.11)	0.121	(0.17)

Source: Original data from an online experiment of professional medical coders in the US, August–September, 2017. * Models control for respondent gender, age in years, race/ethnicity, and years worked as a professional medical coder. † Chart 6 contrasts a female patient to a patient with no identified gender *p < 0.10, **p < 0.05

Results were similar after pooling sub-groups with the same vignettes across the study arms (Table 3), though there were two additional differences: clinical scenarios that identified a female patient with pharyngitis were assigned significantly *higher* average level of service than otherwise identical medical charts that described a male patient (Coef: 0.21, SE: 0.10, *p* < 0.05). Clinical scenarios describing a patient in a wheelchair with hypertension were assigned higher level of service than if the patient was not disabled (Coef: 0.18, SE: 0.10, *p* < 0.10). Results were largely similar when models were weighted with standardized inverse probability weights to account for respondent attrition (Additional file 1: Tables S4 and S5).

**Table 3:** Ordinary least squares regression models of level of service score (range: 1–5) assigned to six medical visit scenarios by professional medical coders, by patient demographic characteristics^*^

	Chart 1		Chart 2		Chart 3		Chart 4		Chart 5		Chart 6	
	Coef	SE	Coef	SE	Coef	SE	Coef	SE	Coef	SE	Coef	SE
Pooled Experimental Arm 1 (Racial Bias)												
White patient or no race identified (ref)												
African-American patient	0.009	(0.10)	0.067	(0.10)	0.114	(0.09)	−0.008	(0.10)	0.088	(0.11)	0.179	(0.13)
Pooled Experimental Arm 2 (Age Bias)												
Middle-aged patient (ref)												
Older adult patient	−0.177	(0.11)	0.199	(0.11)*	0.238	(0.09)**	−0.025	(0.10)	0.076	(0.09)	−0.094	(0.14)
Pooled Experimental Arm 3 (Ability Bias)												
Patient with no disabilities (ref)												
Patient with hearing/visual/physical disability	0.048	(0.11)	0.044	(0.10)	0.117	(0.08)	0.100	(0.10)	0.177	(0.10)*	−0.074	(0.14)
Pooled Experimental Arm 4 (Gender Bias)^†^												
Male patient (ref)												
Female patient	−0.175	(0.10)*	0.015	(0.10)	−0.176	(0.09)**	0.214	(0.10)**	−0.037	(0.10)	0.175	(0.13)
Pooled Experimental Arm 5 (Social Need)												
No social need (ref)												
Any social need	0.097	(0.08)	0.045	(0.08)	0.081	(0.07)	0.009	(0.08)	−0.042	(0.07)	0.023	(0.10)

Source: Original data from an online experiment of professional medical coders in the US, August–September, 2017. This table presents tests that pooled responses across study arms when the clinical scenarios were identical. *Models control for respondent gender, age in years, race/ethnicity, and years worked as a professional medical coder. † Chart 6 contrasts a female patient to a patient with no identified gender *p < 0.10, **p < 0.05

These results should be interpreted in the context of the multiple comparisons conducted. For example, in our primary analyses (Table 2) we found evidence of significant differences in four out of the 30 tests conducted; up to two of these significant tests would have been expected simply due to chance.

## Discussion

Despite the critical role that professional medical coders play in the health care system, little has been published about their coding practices, including the potential influence of bias related to patient race, age, gender, ability, and socioeconomic status on coding decisions. Systematic differences in level of service assigned to medical encounters by patient demographics or social need could translate to substantial revenue disparities, since even subtle differences in coding have been shown in other settings to amount to large sums of money [21]. To our knowledge, this is the first study to evaluate bias in professional medical coders. Overall, results did not reveal that bias plays a substantial role in their coding practices in six common medical conditions.

These findings differed from our a priori expectations that we would see significant differences in coding based on patient demographic factors, including racial/ethnic minority status, and social and economic need [1–10]. Our hypothesis was based on a substantial literature demonstrating bias in many other areas of medicine, [7–10] so the lack of significant findings specific to coders is especially instructive. Rather than indicate that professional coders simply do not have bias, results are more likely to reflect the effectiveness of standardized, professional protocols unique to coding [22] that may serve to counteract implicit bias and that could inform efforts to address bias in other areas of health professional practice [23]. These protocols may serve as a “coder version” of the checklists others have suggested might minimize human error in medicine [24]—in this case the error associated with human bias.

Despite the general lack of significant differences between sub-groups exposed to different versions of the medical encounters, it is important to note the few instances where there were indications of potential bias, including the coding differences based on patient age, with higher level of service assigned to select medical charts describing older versus middle-aged adults. Standard Evaluation & Management (E & M) guidelines indicate that the core components in assigning the level of services are history (e.g. medical, family, and social history), the physical examination, and medical decision-making. It is possible that coders interpreted patients’ older age as a component of medical history and that additional element contributed to higher perceived encounter level of service, though age itself is not part of existing E&M guidelines. The finding that there was higher level of service based on age stands in contrast to other studies of age-related bias in medical decision-making. In those studies, findings have generally shown that elderly patients are less likely to receive treatment for conditions like depression, cancer, and hypertension, [7, 9] which led us to initially hypothesize that these conditions would instead be under-coded.

In two clinical cases — asthma exacerbation and rheumatoid arthritis — female patients were assigned lower level of service codes compared to male patients. These differences are consistent with literature on gender bias in medical decision-making [7, 8, 25]. In general, women are more likely to have physical symptoms discounted and/or interpreted as somatic sequelae of mental health conditions and less likely to receive necessary medical interventions. Medical charts describing a woman with pharyngitis, however, were assigned higher average level of service codes compared to men, suggesting that if there is bias related to gender in coding, the direction may vary by medical condition.

In one case, there was an indication of potential bias based on patient ability: a clinical scenario describing a hypertensive patient in a wheelchair was assigned a higher level of service as compared to an otherwise identical patient with no described ability differences or sensory impairments. However, for five other clinical scenarios related to ability, there were null findings. It may have been that there was some underlying heterogeneity in how coders perceived categories describing a sensory versus physical disability; future research might evaluate the potential for distinct sources of ability-related bias in greater detail. There were also no meaningful differences in average level of service assigned to patients by race or documented social or economic need. Notably, the three domains where we did see some indication of potential bias (age, gender, and ability) cluster into a group associated with eliciting feelings of pity/sympathy from other health professionals [26]. Future work with professional coders could delve further into the complexity of stereotype bias across different populations.

There are important limitations of this study of professional medical coders. First the survey response rate was very low. However, prior research using online recruitment methods have reported response rates at or below 1% [27, 28]. Given that there were no financial incentives offered and that the recruitment invitation was embedded in an organizational newsletter, it is perhaps remarkable that there were nonetheless 596 eligible participants who completed the study. Nevertheless, this leaves open the possibility of selection bias. Second, our results may be further limited in their generalizability given that we excluded some categories of coders (e.g. coders with fewer than three years of experience, coders who work fewer than 15 h per week), although these exclusion criteria were designed to help tease apart coding practices that might be due to inexperience from those that could be driven by implicit bias.

Third, there was participant attrition after participants began the survey; we were left with power sufficient to detect only moderately sized differences in level of service codes between study sub-groups; a larger study might reveal smaller differences that are nonetheless clinically—or financially—significant. Fourth, though we worked with a professional coder association to extract scenarios from national coder exams with some ambiguity in possible answers about level of service, it is possible that there was not sufficient ambiguity in the final clinical scenario describing a patient with chest pain, which would leave little room to detect the influence of other sources of variability. Fifth, the select statistical differences we did observe should be considered in light of multiple comparisons. Sixth, in evaluating ability and social circumstance-related bias, we tested differences in coding in a way that collapsed heterogeneous patient characteristics. For example, coding bias may have been different for patients who were described as homeless as compared to patients described as recently evicted, although these were collapsed into a broader category of ‘housing insecurity’. Finally, the study was not reflective of actual professional medical coder working conditions, which are likely to be more stressful than study conditions. This is particularly important since prior research suggests that implicit bias is exacerbated when other health professionals are pressed for time or face uncertainty in the appropriate diagnosis or course of treatment.7 In practice, professional medical coders who are paid per encounter are indeed subject to time pressure, likely compounded by pressure from performance audits. In one survey fielded by the professional membership organization that supported recruitment for the present study, 82% of nearly 9000 respondents agreed or strongly that their value to their manager is based on accuracy [29]. Future research with professional medical coders is warranted in real life coder practice environments.

## Conclusion

This study is the first to explore whether social bias affects professional medical coders. To our knowledge, it is also the first experimental study done with medical coders on any subject. We found some modest evidence of select differences in average visit level of service assigned to clinical scenarios that varied by patient age (older adult versus middle-aged adult), gender, and ability. However, these results were inconsistent across medical conditions and should be considered in light of the multiple comparisons calculated as part of the analysis. There was otherwise no significant evidence of bias based on other patient characteristics, including race and socioeconomic status. These largely null findings should be replicated in other samples given that they are based on relatively small sample sizes powered to detect only moderately sized effects. However, future research might also explore the possibility that these findings reflect work and training characteristics of professional medical coders that if extrapolated to other health professional practices might help reduce the influence of bias on other health care decision-making.

## Additional file

Additional file 1: **Table S1.** Clinical Scenarios Presented to Professional Medical Coders. **Table S2.** Overview of patient characteristics described in sample clinical scenarios. **Table S3.** Demographic and Work Characteristics. **Table S4.** Mean (SD) level of service score assigned to each medical scenario by randomized group, overall and by patient demographic characteristics or social need. **Table S5.** Ordinary least squares regression models of level of service score (range: 1-5) assigned to six sample charts by professional medical coders, by patient demographic characteristics or social need with stabilized inverse probability weights*. **Table S6.** Ordinary least squares regression models of level of service score (range: 1-5) assigned to six sample charts by professional medical coders, by patient demographic characteristics, with stabilized inverse probability weights*. (DOCX 53 kb)

## Data Availability

The datasets generated and/or analyzed during the current study are not publicly available due to confidentiality restrictions but are available from the corresponding author on reasonable request and pending approval by authors’ and requestors’ institutional review boards.
