# Supplementary material for: An online experiment to assess bias in professional medical coding
Source: BMC Med Inform Decis Mak. 2019 Jun 20;19:115. doi: 10.1186/s12911-019-0832-x (PMC6585065; doi:10.1186/s12911-019-0832-x)
Supplement: Supplementary file 1 — Table S1. Clinical Scenarios Presented to Professional Medical Coders. Table S2. Overview of patient characteristics described in sample clinical scenarios. Table S3. Demographic and Work Characteristics. Table S4. Mean (SD) level of service score assigned to each medical scenario by randomized group, overall and by patient demographic characteristics or social need. Table S5. Ordinary least squares regression models of level of service score (range: 1-5) assigned to six sample charts by professional medical coders, by patient demographic characteristics or social need with stabilized inverse probability weights*. Table S6. Ordinary least squares regression models of level of service score (range: 1-5) assigned to six sample charts by professional medical coders, by patient demographic characteristics, with stabilized inverse probability weights*. (DOCX 53 kb) [file 12911_2019_832_MOESM1_ESM.docx]

**Additional file 1**

| **Table S1. Clinical Scenarios Presented to Professional Medical Coders** | |
| --- | --- |
|  |  |
| **Scenario 1** | A patient presents to the clinic today complaining of cough and difficulty breathing for the last 2-3 days. S/he has not been able to find any of his/her inhalers for the past week. The provider performs an expanded problem focused history and detailed exam.  Assessment/Plan: Asthma exacerbation. Patient was given 125 mg of Solu-Medrol IM today. His/her Advair 250/50 was refilled and s/he was given 2 albuterol inhalers. S/he is instructed to use albuterol 2 puffs every 4 hours while awake for the next week, and then taper it off to p.r.n. If she continues to require albuterol frequently, s/he is to return to the clinic for further evaluation. |
| **Scenario 2** | A patient is seen by an endocrinologist for treatment of newly diagnosed Type 2 diabetes mellitus. The patient's primary care physician has not been able to establish a treatment plan that can adequately control the patient's DM. The endocrinologist performs a comprehensive history and detailed exam. He evaluates the patient's newly diagnosed type 2 diabetes and orders blood glucose levels to continue to be monitored. He orders equipment and provides education for the patient to check their blood glucose at home. He prescribes Humulin. |
| **Scenario 3** | A patient with a new diagnosis of rheumatoid arthritis presents for his/her first evaluation from a rheumatologist. He performs a detailed history and detailed exam. He evaluates the patients newly diagnosed rheumatoid arthritis. He orders an X-ray and prescribes methotrexate injections for the patient that s/he will start receiving in the office. |
| **Scenario 4** | A patient presents to the physician’s office with a cough and sore throat. The physician suspects strep throat and performs a problem focused history and an expanded problem focused exam. The quick strep test results are negative. The patient is diagnosed with pharyngitis. |
| **Scenario 5** | An established patient returns for a follow up appointment to monitor the status of the patient's hypertension. The provider performs an expanded problem focused history and detailed exam. The physician orders routine blood tests and an EKG. The patient's hypertension is stable. The provider requests to see the patient in three months for a follow up appointment. |
| **Scenario 6** | Chief complaint: Chest pain. A patient complains of a substernal chest pain lasting approximately three hours. The ED provider performs a comprehensive history and comprehensive exam.  Intervention: IV: normal saline; Pulse Ox. 97% on room air; The patients 02 was continued. Continuous EKG monitoring established. Nitropaste 2 inches transdermally was applied. PCXR: No acute process. EKG Sinus with no ectopy or ST Changes, interp by me. CMC WBC 9.7, HGB 12.5, HCT 37, PTLS 287, Diff Chem 8:   Na 140, K 3.9, Cl 105, CO2 27, Glucose 166, BUN 13, Creat 0.6, Ca8.8, Ck 46, CKMB 2.3r Troponin: Less than 0.1. PT: 10.6. PTT: 24. INR:  1.02 The patient continued to report no return of his pain during his ER stay. Diagnosis: Chest pain, rule out Ml. Disposition: Admit to CDU. |

| **Table S2. Overview of patient characteristics described in sample clinical scenarios.** | | | | | | | | | | | |
| --- | --- | --- | --- | --- | --- | --- | --- | --- | --- | --- | --- |
|  |  |  |  |  |  |  |  |  |  |  |  |
|  | Arm 1: Racial Bias | | Arm 2: Age Bias | | Arm 3: Ability Bias | | Arm 4: Gender Bias | | Arm 5: Social Needs Bias | | |
|  | Group A | Group B | Group A | Group B | Group A | Group B | Group A | Group B | Group A | Group B | Group C |
| **Chart 1** | 83-year old, female, African-American | 83-year old, female, White | 78-year old, female | 48-year old, female | 83-year old, female, hearing impaired | 83-year old, female | 83-year old, female | 83-year old, male | 83-year old, female, Evicted | 83-year old, female | 83-year old, female, Struggling to Afford Food |
| **Chart 2** | 45-year old, male, African-American | 45-year old, male, White | 75-year old, male | 50-year old, male | 45-year old, male, with wheelchair | 45-year old, male | 45-year old, male | 45-year old, female | 45-year old, male, Homeless | 45-year old, male | 45-year old, male, Cannot Afford Groceries |
| **Chart 3** | 53-year old, female, African-American | 53-year old, female, White | 83-year old, female | 38-year old, female | 53-year old, female, visual impairment | 53-year old, female | 53-year old, female | 53-year old, male | 53-year old, female, Struggling to Afford Food | 53-year old, female, Evicted | 53-year old, female |
| **Chart 4** | 80-year old, female, White | 80-year old, female, African-American | 42-year old, female | 78-year old, female | 80-year old, female | 80-year old, hearing impairment | 80-year old, male | 80-year old, female | 80-year old, female, Cannot Afford Groceries | 80-year old, female, Homeless | 80-year old, female |
| **Chart 5** | 47-year old, male, White | 47-year old, male, African-American | 50-year old male | 83-year old, male | 47-year old, male | 47-year old, male, wheelchair | 47-year old, male | 47-year old, female | 47-year old, male | 47-year old, male, Homeless | 47-year old, male, Struggling to Afford Food |
| **Chart 6** | 53-year old, White | 53-year old, African-American | 38-year old, male | 75-year old, male | 53-year old | 53-year old, visual impairment | 53-year old | 53-year old, female | 53-year old | 53-year old, Cannot Afford Groceries | 53-year old, Homeless |
| **Note: Gridlines are placed around charts that presented identical clinical scenarios and patient demographic characteristics. In ancillary analyses, we pooled responses from across study arms for identical clinical scenarios.** | | | | | | | | | | | |

| **Table S3. Demographic and Work Characteristics** | | | | | | | | | | | | | | | |
| --- | --- | --- | --- | --- | --- | --- | --- | --- | --- | --- | --- | --- | --- | --- | --- |
|  |  |  |  |  |  |  |  |  |  |  |  |  |  |  |  |
|  | Experimental Group and Subgroup Assignment | | | | | | | | | | | |  | | |
|  | Racial Bias | | Age Bias | | Ability Bias | | Gender Bias | | Social Need Bias | | | |  | |  |
|  | 1 (N=49) | 2 (N=50) | 3 (N=52) | 4 (N=58) | 5 (N=57) | 6 (N=54) | 7 (N=51) | 8 (N=57) | 9 (N=49) | 10 (N=48) | 11 (N=58) | **Test of Significant Difference** | |  |  |
| **Gender (N=583)** |  |  |  |  |  |  |  |  |  |  |  |  |  |  |  |
| Female | 43 (87.8) | 49 (98.0) | 49 (94.2) | 53 (91.4) | 46 (80.7) | 52 (96.3) | 47 (92.2) | 52 (91.2) | 46 (93.9) | 45 (93.8) | 56 (96.6) |  | |  |  |
| Male | 6 (12.2) | 1 (2.0) | 2 (3.9) | 5 (8.6) | 11 (19.3) | 2 (3.7) | 3 (5.9) | 5 (8.8) | 3 (6.1) | 3 (6.3) | 2 (3.4) |  | |  |  |
| Other | 0 (0.0) | 0 (0.0) | 1 (1.9) | 0 (0.0) | 0 (0.0) | 0 (0.0) | 1 (2.0) | 0 (0.0) | 0 (0.0) | 0 (0.0) | 0 (0.0) | Chi2 = 28.9; p=0.089 | |  |  |
| **Race/Ethnicity (N=550)** | (N=48) | (N=46) | (N=46) | (N=56) | (N=55) | (N=50) | (N=47) | (N=53) | (N=48) | (N=44) | (N=57) |  | |  |  |
| Non-Latino White | 41 (85.4) | 37 (80.4) | 40 (87.0) | 42 (75.0) | 41 (74.6) | 44 (88.0) | 40 (85.1) | 40 (75.5) | 41 (85.4) | 36 (81.8) | 46 (80.7) |  | |  |  |
| Racial/Ethnic Minority/Non-White | 7 (14.6) | 9 (19.6) | 6 (13.0) | 14 (25.0) | 14 (25.4) | 6 (12.0) | 7 (14.9) | 13 (24.5) | 7 (14.6) | 8 (18.2) | 11 (19.3) | Chi2 = 8.34; p=0.595 | |  |  |
| **Age (N=570), mean (SD)** | 48.3 (10.8) | 46.5 (11.6) | 43.8 (10.5) | 46.7 (11.1) | 42.5 (10.6) | 47.1 (10.3) | 45.6 (10.9) | 44.4 (11.5) | 47.1 (10.8) | 47.1 (12.3) | 43.7 (12.2) | F=1.44; p=0.158 | |  |  |
| **Years worked (N=580), mean (SD)** | 13.1 (9.2) | 10.5 (8.2) | 12.5 (10.5) | 10.6 (8.1) | 10.3 (7.0) | 12.8 (8.5) | 9.8 (8.9) | 10.7 (7.9) | 13.7 (10.2) | 11.4 (9.1) | 10.9 (9.5) | F=1.14; p=0.330 | |  |  |
| **Hours per week (N=577), mean (SD)*** | 36.0 (12.8) | 38.5 (7.6) | 37.9 (14.7) | 38.9 (10.6) | 37.6 (11.4) | 38.2 (8.3) | 38.9 (11.7) | 34.5 (12.0) | 36.2 (13.0) | 39.7 (8.2) | 38.0 (12.8) | F=0.94; p=0.493 | |  |  |
| Source: Original data from an online experiment of professional medical coders in the US, August - September, 2017. | | | | | | | | | | | | | | | |

| **Table S4. Mean (SD) level of service score assigned to each medical scenario by randomized group, overall and by patient demographic characteristics or social need** | | | | | | | | | | | | | |
| --- | --- | --- | --- | --- | --- | --- | --- | --- | --- | --- | --- | --- | --- |
|  |  | Chart 1 | | Chart 2 | | Chart 3 | | Chart 4 | | Chart 5 | | Chart 6 | |
|  |  | Mean | (SD) | Mean | (SD) | Mean | (SD) | Mean | (SD) | Mean | (SD) | Mean | (SD) |
| **Experimental Arm 1 (Racial Bias)** | | 3.40 | (0.60) | 3.36 | (0.65) | 3.09 | (0.60) | 2.55 | (0.58) | 3.09 | (0.61) | 4.48 | (0.63) |
|  | White patient | 3.38 | (0.70) | 3.31 | (0.59) | 3.15 | (0.56) | 2.57 | (0.58) | 3.05 | (0.57) | 4.47 | (0.67) |
|  | African-American patient | 3.43 | (0.50) | 3.38 | (0.70) | 3.02 | (0.64) | 2.54 | (0.59) | 3.13 | (0.66) | 4.49 | (0.59) |
| **Experimental Arm 2 (Age Bias)** | | 3.52 | (0.70) | 3.36 | (0.65) | 3.12 | (0.57) | 2.58 | (0.57) | 3.04 | (0.46) | 4.28 | (0.87) |
|  | Middle-aged patient | 3.55 | (0.68) | 3.26 | (0.68) | 2.98 | (0.57) | 2.61 | (0.61) | 3.09 | (0.56) | 4.34 | (0.78) |
|  | Older adult patient | 3.48 | (0.73) | 3.47 | (0.62) | 3.28 | (0.54) | 2.56 | (0.54) | 3.00 | (0.34) | 4.23 | (0.95) |
| **Experimental Arm 3 (Ability Bias)** | | 3.40 | (0.65) | 3.32 | (0.64) | 3.11 | (0.58) | 2.73 | (0.55) | 3.10 | (0.58) | 4.30 | 0.769 |
|  | Patient without disabilities | 3.47 | (0.57) | 3.34 | (0.56) | 3.08 | (0.53) | 2.70 | (0.60) | 3.02 | (0.61) | 4.30 | (0.68) |
|  | Patient with disability | 3.33 | (0.72) | 3.31 | (0.72) | 3.13 | (0.62) | 2.75 | (0.48) | 3.20 | (0.54) | 4.30 | (0.87) |
| **Experimental Arm 4 (Gender Bias)*** | | 3.39 | (0.67) | 3.34 | (0.59) | 3.15 | (0.54) | 2.44 | (0.54) | 3.00 | (0.59) | 4.38 | (0.76) |
|  | Male patient | 3.48 | (0.68) | 3.37 | (0.57) | 3.27 | (0.56) | 2.38 | (0.57) | 3.00 | (0.56) | 4.35 | (0.75) |
|  | Female patient | 3.27 | (0.63) | 3.32 | (0.61) | 3.02 | (0.48) | 2.51 | (0.51) | 3.00 | (0.62) | 4.40 | (0.78) |
| **Experimental Arm 5 (Social Need)** | | 3.45 | (0.60) | 3.33 | (0.60) | 3.12 | (0.42) | 2.60 | (0.53) | 2.99 | (0.47) | 4.30 | (0.78) |
|  | No social need | 3.35 | (0.67) | 3.26 | (0.57) | 3.08 | (0.38) | 2.58 | (0.53) | 2.98 | (0.42) | 4.35 | (0.68) |
|  | Housing Insecurity | 3.49 | (0.62) | 3.34 | (0.62) | 3.11 | (0.44) | 2.64 | (0.53) | 3.04 | (0.53) | 4.29 | (0.74) |
|  | Food Insecurity | 3.49 | (0.54) | 3.38 | (0.62) | 3.18 | (0.45) | 2.58 | (0.55) | 2.95 | (0.43) | 4.26 | (0.91) |
| Source: Original data from an online experiment of professional medical coders in the US, August - September, 2017. * Chart 6 contrasts a female patient to a patient with no identified gender. | | | | | | | | | | | | | |

| **Table S5. Ordinary least squares regression models of level of service score (range: 1-5) assigned to six sample charts by professional medical coders, by patient demographic characteristics or social need with stabilized inverse probability weights*** | | | | | | | | | | | | | | | | | | | | | | | | | | | |  |
| --- | --- | --- | --- | --- | --- | --- | --- | --- | --- | --- | --- | --- | --- | --- | --- | --- | --- | --- | --- | --- | --- | --- | --- | --- | --- | --- | --- | --- |
|  |  |  |  |  |  |  |  |  |  |  |  |  |  |  |  |  |  |  |  |  |  |  |  |  |  |  |  |  |
|  |  | | Chart 1 | | | | Chart 2 | | | | Chart 3 | | | | Chart 4 | | | | Chart 5 | | | | Chart 6 | | | | | |
|  |  | | Coef | | SE | | Coef | | SE | | Coef | | SE | | Coef | | SE | | Coef | | SE | | Coef | | SE | | | |
| **Experimental Arm 1 (Racial Bias)** | |  | |  | |  | |  | |  | |  | |  | |  | |  | |  | |  | |  | |  |  |  |
|  | White patient (ref) | |  | |  | |  | |  | |  | |  | |  | |  | |  | |  | |  | |  | | | |
|  | African-American patient | | -0.100 | | (0.13) | | 0.157 | | (0.16) | | 0.130 | | (0.13) | | 0.034 | | (0.13) | | 0.037 | | (0.14) | | 0.098 | | (0.16) | | | |
| **Experimental Arm 2 (Age Bias)** | |  | |  | |  | |  | |  | |  | |  | |  | |  | |  | |  | |  | |  |  |  |
|  | Middle-aged patient (ref) | |  | |  | |  | |  | |  | |  | |  | |  | |  | |  | |  | |  | | | |
|  | Older adult patient | | -0.032 | | (0.13) | | 0.321 | | (0.14)** | | 0.315 | | (0.13)** | | -0.101 | | (0.14) | | -0.146 | | (0.10) | | -0.114 | | (0.20) | | | |
| **Experimental Arm 3 (Ability Bias)** | |  | |  | |  | |  | |  | |  | |  | |  | |  | |  | |  | |  | |  |  |  |
|  | Patient without disabilities (ref) | |  | |  | |  | |  | |  | |  | |  | |  | |  | |  | |  | |  | | | |
|  | Patient with hearing/visual/physical disability | | 0.016 | | (0.12) | | 0.082 | | (0.13) | | 0.055 | | (0.11) | | 0.031 | | (0.11) | | 0.116 | | (0.12) | | 0.000 | | (0.17) | | | |
| **Experimental Arm 4 (Gender Bias)^†^** | |  | |  | |  | |  | |  | |  | |  | |  | |  | |  | |  | |  | |  |  |  |
|  | Male patient (ref) | |  | |  | |  | |  | |  | |  | |  | |  | |  | |  | |  | |  | | | |
|  | Female patient | | -0.263 | | (0.15)* | | -0.101 | | (0.14) | | -0.200 | | (0.11)* | | 0.131 | | (0.12) | | -0.011 | | (0.14) | | 0.145 | | (0.18) | | | |
| **Experimental Arm 5 (Social Need)** | |  | | | |  | | | |  | | | |  | | | |  | | | |  | | | | |  |  |
|  | No social need (ref) | |  | |  | |  | |  | |  | |  | |  | |  | |  | |  | |  | |  | | | |
|  | Housing Insecurity | | 0.121 | | (0.13) | | 0.056 | | (0.12) | | 0.105 | | (0.09) | | -0.112 | | (0.12) | | -0.045 | | (0.10) | | 0.048 | | (0.18) | | | |
|  | Food Insecurity | | 0.076 | | (0.12) | | 0.045 | | (0.10) | | 0.026 | | (0.09) | | 0.028 | | (0.11) | | 0.018 | | (0.10) | | 0.114 | | (0.15) | | | |
| Source: Original data from an online experiment of professional medical coders in the US, August - September, 2017. *Models control for respondent gender, age in years, race/ethnicity, and years worked as a professional medical code. †Chart 6 contrasts a female patient to a patient with no identified gender. *p<0.10, **p<0.05. | | | | | | | | | | | | | | | | | | | | | | | | | | | |  |

| **Table S6. Ordinary least squares regression models of level of service score (range: 1-5) assigned to six sample charts by professional medical coders, by patient demographic characteristics, with stabilized inverse probability weights*** | | | | | | | | | | | | | |
| --- | --- | --- | --- | --- | --- | --- | --- | --- | --- | --- | --- | --- | --- |
|  |  |  |  |  |  |  |  |  |  |  |  |  |  |
|  |  | Chart 1 | | Chart 2 | | Chart 3 | | Chart 4 | | Chart 5 | | Chart 6 | |
|  |  | Coef | SE | Coef | SE | Coef | SE | Coef | SE | Coef | SE | Coef | SE |
| **Mixed Experimental Group 1 (Racial Bias)** | |  |  |  |  |  |  |  |  |  |  |  |  |
|  | White patient or no race identified (ref) |  |  |  |  |  |  |  |  |  |  |  |  |
|  | African-American patient | 0.005 | (0.09) | 0.090 | (0.12) | 0.134 | (0.10) | 0.003 | (0.10) | 0.121 | (0.12) | 0.171 | (0.12) |
| **Mixed Experimental Group 2 (Age Bias)** | |  |  |  |  |  |  |  |  |  |  |  |  |
|  | Middle-aged patient (ref) |  |  |  |  |  |  |  |  |  |  |  |  |
|  | Older adult patient | -0.201 | (0.11)* | 0.252 | (0.12)** | 0.262 | (0.10)** | -0.101 | (0.14) | -0.026 | (0.07) | -0.078 | (0.16) |
| **Mixed Experimental Group 3 (Ability Bias)** | |  |  |  |  |  |  |  |  |  |  |  |  |
|  | Patient with no disabilities (ref) |  |  |  |  |  |  |  |  |  |  |  |  |
|  | Patient with hearing/visual/physical disability | 0.074 | (0.10) | 0.037 | (0.11) | 0.127 | (0.09) | 0.115 | (0.08) | 0.183 | (0.10) | -0.069 | (0.15) |
| **Mixed Experimental Group (Gender Bias)^†^** | |  |  |  |  |  |  |  |  |  |  |  |  |
|  | Male patient (ref) |  |  |  |  |  |  |  |  |  |  |  |  |
|  | Female patient | -0.188 | (0.11)* | 0.008 | (0.11) | -0.183 | (0.09)** | 0.192 | (0.10)** | -0.020 | (0.11) | 0.163 | (0.14) |
| **Mixed Experimental Group (Social Need)** | |  |  |  |  |  |  |  |  |  |  |  |  |
|  | No social need (ref) |  |  |  |  |  |  |  |  |  |  |  |  |
|  | Any social need | 0.163 | (0.09)* | 0.021 | (0.08) | 0.088 | (0.06) | 0.013 | (0.08) | -0.032 | (0.07) | 0.007 | (0.10) |
| Source: Original data from an online experiment of professional medical coders in the US, August - September, 2017. *Models control for respondent gender, age in years, race/ethnicity, and years worked as a professional medical coder. † Chart 6 contrasts a female patient to a patient with no identified gender. *p<0.10, **p<0.05. | | | | | | | | | | | | | |
